# Effect of the National Enhanced Service for weight management on the content of annual review consultations for patients living with obesity and hypertension and/or diabetes

**DOI:** 10.1111/cob.12718

**Published:** 2024-11-10

**Authors:** Stella J. P. Haffner, Sarah Mounsey, Rachna Begh, Anisa Hajizadeh, Alice E. Hobson, Paul Doody, Charlotte Albury, Suzanne Mara, Laura Heath, Kayley McPherson, Susan A. Jebb, Paul Aveyard

**Affiliations:** ^1^ Nuffield Department of Primary Care Health Sciences University of Oxford Oxford UK; ^2^ NIHR Oxford and Thames Valley Applied Research Collaboration University of Oxford Oxford UK; ^3^ School of Geography and the Environment University of Oxford Oxford UK; ^4^ Discipline of Public Health and Primary Care, Institute of Population Health, School of Medicine, Trinity College Dublin the University of Dublin Ireland; ^5^ NIHR Oxford Biomedical Research Centre John Radcliffe Hospital Oxford UK; ^6^ NIHR Oxford Health Biomedical Research Centre Warneford Hospital Oxford UK

**Keywords:** diabetes, hypertension, National Enhanced Service, obesity, primary care, weight management

## Abstract

Guidelines specify that clinicians should support patients living with obesity by referring to weight management programmes (WMPs), but clinicians do so infrequently. To provide additional support to patients living with obesity and weight‐related conditions, the UK government instated the National Enhanced Service (NES) for weight management in England, including a reimbursement to general practices for referring eligible patients to WMPs. To assess the impact of the NES on conversations regarding weight and relevant behavioural risk factors in primary care consultations we recruited 11 medical practices in England where the NES was operating and six comparator practices from Scotland and Wales where the NES was not implemented. Clinicians audio‐recorded annual review appointments of patients living with obesity and hypertension and/or diabetes. The content of these consultations was synthesised using quantitative content analysis. Consultations with 92 patients were analysed: 58 in England and 34 in Scotland and Wales. No difference was found between the NES sites (England) and non‐NES sites (Scotland and Wales) in the proportion of referrals made to WMPs. Clinicians in England weighed patients and took other body measurements more often, mentioned body mass index more often, and had more detailed discussions about patients' diets, but there was no evidence that they differed in their discussion of WMPs or other modifiable risk factors. We found no strong evidence that the NES affected how clinicians addressed weight management or related behavioural risk factors within annual review consultations for patients living with obesity and hypertension and/or diabetes.

**What is already known about this subject**

- Although clinical guidelines recommend offering patients living with obesity or high waist circumference support with weight management, clinicians rarely offer referrals to weight management programmes.
- Incentive payment schemes, such as the Quality and Outcomes Framework, have demonstrated potential to increase the incidence of intervention within primary care by rewarding practices when clinicians address specific risk factors.
- In 2021, the UK government instated the National Enhanced Service to encourage quality of care in the management of excess weight and obesity, reimbursing practices with £11.50 for each patient living with obesity referred to a weight management programme.

**What this study adds**

- The National Enhanced Service did not impact the provision of support for weight management in annual review consultations for patients living with obesity and hypertension and/or diabetes.
- However, clinicians under the National Enhanced Service tended to take body measures (e.g., weight and waist circumference) more often than clinicians not part of the National Enhanced Service, and they also tended to have more detailed conversations about diet with their patients.

## 1 INTRODUCTION

UK clinical guidelines recommend that health care professionals (HCPs) offer patients living with obesity additional support – such as a referral to a weight management programme (WMP).^1^ Despite this, HCPs infrequently offer referrals to weight management services,^2^ citing several barriers to delivering interventions for weight management, including time pressures, the perception that patients living with obesity may not be interested in or motivated to lose weight, a lack of confidence in treatment options, and uncertainty in how best to raise the topic of weight.^3^, ^4^, ^5^, ^6^, ^7^ However, evidence suggests that brief interventions such as an opportunistic recommendation to try a weight management programme are effective. A trial recruited patients attending routine primary care appointments and randomly allocated them to a control group where their practitioner advised them to lose weight for health reasons or to an intervention group where their practitioner endorsed, offered, and facilitated enrolment in a WMP free of user cost.^8^ The intervention was highly acceptable to clinicians and patients, and uptake of support was four times higher in the intervention group than the control group, leading to significantly greater weight loss at 1 year. Further economic analysis showed that the intervention would be cost‐saving for health systems within 10 years of its introduction.^9^

Incentive payment schemes have the potential to increase the incidence of intervention within primary care by financially rewarding practices when HCPs address specific risk factors. Previous financial incentive schemes such as the Quality and Outcomes Framework (QOF) have positively impacted the rate of referral in the case of smoking and quality of care for people with diabetes,^10^, ^11^ suggesting that a similar scheme addressing obesity could change the frequency and quality of interventions for weight loss. In September 2021, the government instated the National Enhanced Service for weight management (NES) in England. This initiative introduced a new national WMP for people living with obesity and either hypertension and/or diabetes and encouraged practices to take a comprehensive approach to intervening on obesity. Practices were reimbursed with £11.50 for each patient referred to a WMP who had a body mass index (BMI) of ≥30 kg/m^2^ if of white ethnicity or ≥27.5 kg/m^2^ for all other ethnic groups.

The aim of the NES was to increase the likelihood of discussion of weight and weight management in consultations between HCPs and patients living with obesity, to normalise those conversations, and to create a supportive infrastructure in practices for patients living with excess weight and obesity. However, the incentive payment was offered for a completed referral for patients living with obesity, not for having a discussion around weight management and not for attending a WMP. Nowadays, practices have several mechanisms to increase referrals and thereby receive payments, including offering referrals by text message. It is therefore possible for practices to respond to the NES by increasing referrals and thereby receive payments but not meaningfully meet the overall aim of the NES.

We examined the impact of the NES on HCPs' behaviour, focusing on consultations where weight management discussions might be expected to occur. Given the focus of the NES on providing weight management behavioural support, we expected referrals to WMPs to increase. Likewise, referral‐relevant behaviours (e.g., weighing the patient) were expected to increase, as HCPs are required to have recent height and weight data to make a referral. Mentions of other behavioural risk factors were recorded. It is plausible that, if HCPs are prompted by the NES to broach the topic of weight, HCPs and/or patients may raise other behavioural risk factors too. However, it is also possible that taking time to discuss weight issues could ‘*squeeze out*’ discussion of other behavioural risk factors, given time constraints in primary care. Finally, because HCPs perceive a connection between obesity and mental health, particularly the risk of upsetting a patient by discussing weight management,^6^ mentions of mental health and wellbeing were also assessed.

To examine the impact of the NES on primary care consultations, we recorded annual review consultations for patients with hypertension and/or type 2 diabetes who were living with obesity, conditions where weight loss is part of the recommended care, and where HCPs might be expected to make a referral to a WMP. We explained the purpose of the study in general terms only, meaning that neither the patients nor practitioners were aware of the focus on weight‐related discussion, and we documented referral to all WMPs regardless of type or sponsor. We aimed to explore the impact of the NES on conversations about weight and other behavioural risk factors by comparing consultations in England, where the NES was operating, to Scotland and Wales, where it was not.

## 2 METHODS

Ethical approval was obtained from the Northern Ireland Research Ethics Committee, IRAS reference 293 284, on 25 February 2021. The original design presented in the protocol specified that the impact of the NES would be examined by comparing consultations in England to those in Scotland and Wales before the NES was introduced and after its introduction to assess the change that occurred in England. We aimed to recruit approximately five practices in each area to record five consultations each before and after the introduction of the NES. However, delays in the approval and recruitment process meant that the NES was already operating before we could record consultations for the baseline assessment. Instead, we increased our target to six recordings per practice and collected data after the introduction of the NES, comparing English sites with Scottish and Welsh sites to assess the impact of the NES.

We worked with the Clinical Research Network (CRN) to recruit practices in each of our regions of interest. The CRN sent out a study advertisement to all research‐active practices, and interested practices had the opportunity to opt into the study. Participating practices searched their electronic records for patients with a BMI ≥ 30 kg/m^2^ if of white ethnicity or ≥27.5 kg/m^2^ for all other ethnic groups, who had diabetes and/or hypertension, and were due for an annual review. Each practice sent potential participants an invitation to participate, recruiting a total of six patients per practice. Both HCPs and patients were told that the study focused on the discussion of lifestyle behaviours in general without revealing the specific focus on weight management to avoid distorting normal practice, and all participants were debriefed on the true nature of the study after participating. Consultations took place either by telephone, video, or face‐to‐face. Each HCP recorded their consultations on a secure device and used a secure network to upload them for analysis.

All recordings were anonymised; patient age, gender, and ethnicity were not known by the research team and HCP details were also excluded. The content of the consultation recordings was synthesised using quantitative content analysis.^12^ We created an a priori coding scheme focusing on specific elements from the discussion of behavioural risk factors related to obesity, hypertension and/or diabetes, and weight management. For example, our coding scheme included categories to capture how HCPs took body measurements, how patients responded to the topics raised in the consultations, and how HCPs offered lifestyle management support (Appendix S1). As we listened to the recordings, the coding scheme was adapted to account for emergent conversation topics, particularly to capture the nuance of how weight management referrals were made. Two assessors listened to the consultations for each element in the coding scheme. From the coding scheme, we calculated three values: (1) the total counts for each conversation element, (2) the frequency of each conversation element within a region by dividing the English total counts by the English sample size and the comparator total counts by the comparator sample size, and (3) the 95% confidence intervals for the difference in frequency between practices in England (which had the NES) and practices in Scotland and Wales (where there was no NES). By comparing key elements of the annual review consultations, focusing on the discussion of weight and relevant risk factors, this analysis aimed to explore the impact of the NES on the way HCPs address the topic of weight management and provide referrals to WMPs.

## 3 RESULTS

### 3.1 Recruitment

Eleven practices were recruited from England where the NES was operating and six comparator practices from Scotland and Wales where there was no NES. Ninety‐two patients with obesity and a diagnosis of diabetes and/or hypertension participated in the study: 58 in England and 34 in Scotland and Wales. Recordings took place between June 2021 and April 2024.

### 3.2 HCP referral behaviour and discussion of WMPs

**FIGURE 1:**
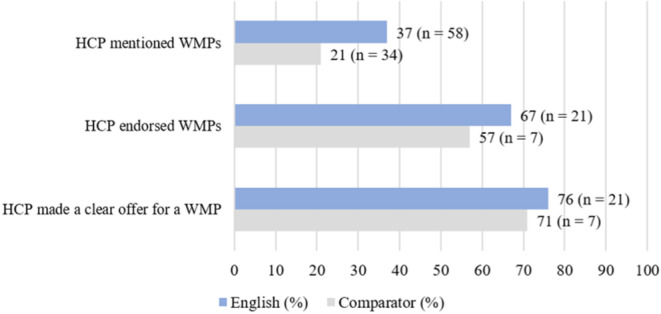
Summary of how HCPs discussed WMPs.

There was no difference between referral rates and how WMPs were discussed in NES sites and non‐NES sites. WMPs were mentioned in 37% of all consultations in England and 21% in comparator sites, a difference of 16% (95% CI −2.8 to 34). As a percentage of all consultations where WMPs were mentioned, WMPs were endorsed by HCPs in 67% of English consultations and 57% of comparator consultations, a difference of 10% (95% CI −32 to 51). Of the consultations where WMPs were mentioned, a referral was offered to patients in 76% of English consultations and 71% of comparator consultations, a difference of 5% (95% CI −3 to 43) (Figure 1).

There was no evidence that the NES changed the nature of the conversation about WMPs, which were discussed in 30% of all consultations. HCPs in England and comparator regions had similar approaches to discussing WMPs, usually commenting on whether the programme was in‐person or online, whether the programme was local, whether the programme had a personal aspect such as one‐on‐one coaching, and whether the patient would have to pay for the programme. If the HCP endorsed the programme, they might emphasise the social aspect of attending a WMP, explain that the programme could help alleviate diabetes symptoms, or mention that other patients have had a positive experience.

### 3.3 Referral‐relevant behaviour

**FIGURE 2:**
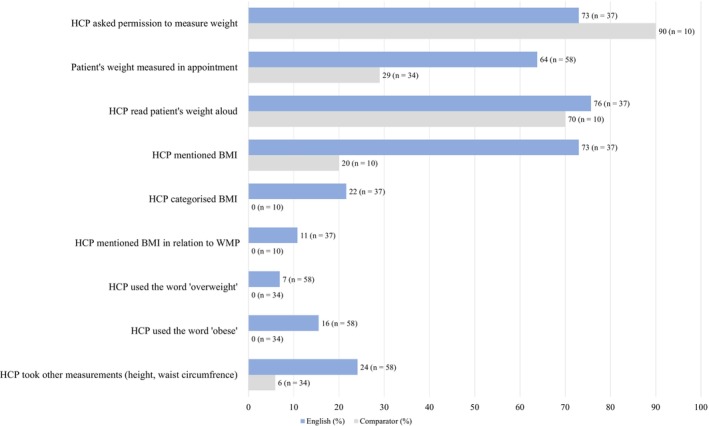
Summary of discussion elements around body measuring in annual review appointments.

To make a referral, HCPs need to include a recent height and weight measurement for their patients. Overall, the annual review consultations in England demonstrated a greater focus on body measurements. Compared to Scotland and Wales, 35% (95% CI 15 to 54) more patients were weighed in England, additional body measures (height and waist circumference) were taken 18% (95% CI 4.7 to32) more often, and BMI was mentioned 53% (95% CI 24–82) more frequently. There were additional differences in the way body measures were discussed. HCPs in England sometimes described a patient's weight using the World Health Organization BMI categories (i.e., ‘in the obese range’), whereas HCPs in comparator sites never categorised BMI, never mentioned BMI in relation to a WMP, and never used the words ‘overweight’ or ‘obese’ during their consultations (Figure 2).

### 3.4 Patient response to referral and discussion of WMPs

When HCPs in both England and comparator sites clearly offered a referral to a WMP, patients responded in one of three ways: they accepted, they declined, or they neither accepted nor declined. Too few patients were offered a referral to formally assess whether there were differences in responses between England and comparator sites; however, 7 of 16 patients accepted an offer of referral in England and all four patients did so in the comparator sites (Table 1). Notably, patients in England and in the comparator sites offered similar thoughts when responding to the topic of weight management, regardless of whether a formal referral was made. Commonly, patients said that they were not interested in attending a WMP because they already knew what they ‘should be doing’ or expressed concerns about the value of attending a WMP: ‘I know how to lose weight. I know what I should and shouldn't eat. I just think – at my age – is it worth it?’ In contrast, two patients in England explicitly expressed that they wanted help to lose weight but were not offered a referral.

**TABLE 1:** Summary of weight and weight management‐related discussion elements during annual review appointment.

Content	English frequency/total sample	English %	Comparator frequency/total sample	Comparator %	Absolute difference in % (95% CI)^a^
Weight measured in appointment	37/58	64	10/34	29	35 (15–54)
HCP asked permission before measuring weight	27/37	73	9/10	90	−17 (−40 to 6.4)
HCP read patient's weight aloud	28/37	76	7/10	70	6 (−26 to 37)
HCP mentions BMI	27/37	73	2/10	20	53 (24–82)
HCP categorised BMI	8/37	22	0/10	0	–
HCP mentioned BMI in relation to WMP	4/37	11	0/10	0	–
HCP compared weight to previous measurement	22/37	59	4/10	40	19 (−15 to 54)
HCP used the word ‘overweight’	4/58	7	0/34	0	–
HCP used the word ‘obese’	9/58	16	0/34	0	–
HCP took other measurements (height or waist circumference)	14/58	24	2/34	6	18 (4.7–32)
HCP mentioned WMP	21/58	37	7/34	21	16 (−2.8 to 34)
HCP endorsed WMP	14/21	67	4/7	57	10 (−32 to 51)
HCP made a clear offer for a WMP	16/21	76	5/7	71	5 (−33 to 43)
HCP gave space for patient to accept or refuse referral	16/16	100	4/5	80	20 (−15 to 55)
Patient accepted referral	7/16	44	4/4	100	−56 (−81 to −32)
Patient declined referral	5/16	31	0/4	0	–
Patient neither accepted nor declined referral	4/16	25	0/4	0	–
HCP questioned patient's reasons for declining	1/5	20	–	–	–
HCP accepted patient's refusal of referral	4/5	80	–	–	–

^a^ Absolute difference calculated as English percentage minus comparator percentage.

### 3.5 Discussion of modifiable risk factors

**FIGURE 3:**
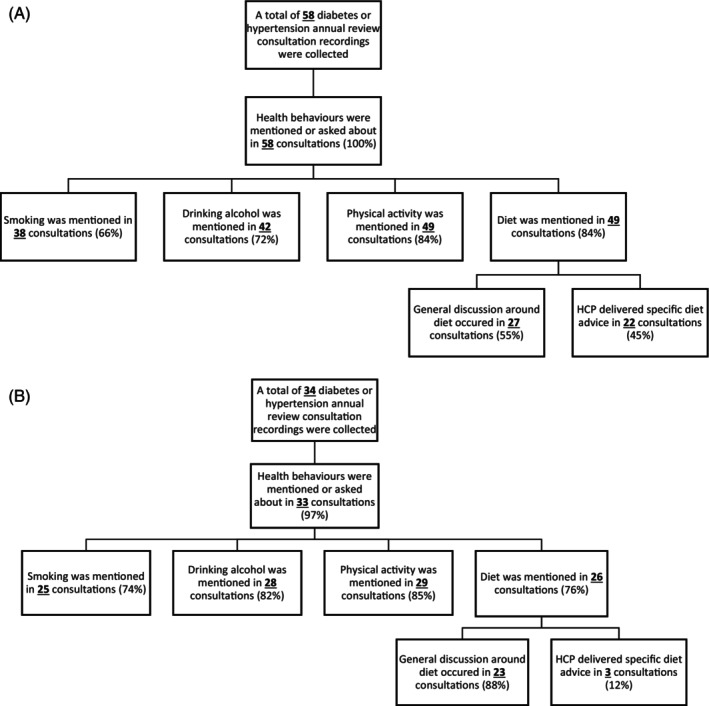
(A) Summary of discussions on behavioural risk factors in English NES practices. (B) Summary of discussions on behavioural risk factors in Scottish and Welsh comparator practices without the NES.

There was no evidence that the NES changed the frequency of discussions about diet, physical activity, drinking, or smoking (Figure 3A,B). When diet was discussed during consultations, HCPs in comparator sites typically had general, non‐specific conversations about the patient's diet (i.e., briefly describing what makes a healthy diet in 88% of consultations). Meanwhile, 45% of English consultations had more detailed conversations around diet (e.g., explaining how carbohydrates affect blood glucose, describing foods that contribute positively to heart health, providing example meals that patients could try, and encouraging patients to identify foods for which they might reduce their consumption), a difference of 23% (95% CI 5.6 to 42).

### 3.6 Mental health conversations

In England, mental health was mentioned during 17 of 58 (29%) appointments and was mentioned in comparator sites during 5 of 34 (15%) appointments, a difference of 14% (95% CI −2.1 to 31), with most of these conversations initiated by patients. HCPs rarely explicitly enquired about mental health, compared with enquiries about behavioural risk factors such as drinking and smoking. Despite this, patients created opportunities to discuss poor mental health in relation to weight management, alluding to life‐related stress when asked about their weight and modifiable behavioural risk factors, for instance explaining that bereavement had affected their eating behaviour.

## 4 DISCUSSION

We found that conversations about weight and behavioural risk factors during annual review consultations were largely similar in England, where the NES operated, compared to Scotland and Wales, where there was no NES. Despite the presence of the NES with its incentive for referral, there was no evidence of a clear difference in the rate of referral to WMPs, with referrals being infrequent in both England and the comparator sites. Where differences emerged, there was evidence that consultations in England had a greater focus on body measures and that, relative to the comparator sites, HCPs in England more frequently used the time provided by annual review consultations to educate their patients on healthy eating.

While the NES aimed to increase the discussion of weight and weight management within consultations, the lack of an effect observed here highlights that we do not yet understand how financial incentives affect HCP behaviour. Reviews examining the effect of financial incentive schemes are mixed, showing modest positive but uncertain effects of financial incentives.^13^, ^14^, ^15^ Meanwhile, individual studies on smoking behaviour and diabetes care have demonstrated that financial incentives delivered through the QOF have positively impacted the rate of referral in the case of smoking and quality of care for people with diabetes.^10^, ^11^ The success of a financial incentive like the QOF and the lack of impact for an incentive like the NES may be explained by loss aversion. When the QOF was introduced, a portion of the money that had gone to GPs by right was removed and given ‘back’ if GPs met certain targets for performance. Thus, failure to meet the QOF targets, where most practices achieve most targets, is perceived as a financial loss. In contrast, the NES offers an opportunity to achieve additional income through additional work. This incentive may not be as motivating as the QOF because the cognitive bias of loss aversion means that money gained is not as salient as money lost.^16^

Although we found no strong evidence that the NES and its incentive affected the referrals to weight management services within annual review consultations, the relatively small sample and wide confidence intervals reported here mean it is not possible to conclude that the NES had no impact, particularly if the effect was small. The data collection period of the present study, taking place during the first two years of the NES between July 2021 and July 2023, means that this investigation is limited in its ability to comment on time‐varying differences in the effect of the NES. On the one hand, due to per‐practice quotas put in place by the integrated care boards, each practice has a maximum number of patients they can refer to a WMP and receive reimbursement for. It is possible that the practices we sampled had already reached their quotas by the time data were collected; although this is unlikely given that the data were collected soon after the instatement of the NES. Likewise, because quotas directly limit the reimbursement for referral, not the referral itself, in order for quotas to affect the present findings, we would have to assume that HCPs avoided making referrals if they knew they were no longer being reimbursed. On the other hand, the two‐year data collection period means that this investigation is limited in its ability to comment on the impact of the NES if it becomes more integrated within general practice over time. It may be that a policy targeting the provision of weight management support would take longer to influence general practice than new guidelines intervening, for instance, on smoking behaviour because weight is considered a particularly sensitive issue within primary care. Indeed, the lack of intervention observed in the present study is consistent with previous work showing that HCPs have concerns about discussing weight with patients and evidence that advice is given to only around 4%–6% of people living with obesity each year, with referrals in 2%–8% per year, depending upon BMI category.^2^, ^17^ Reluctance to address the topic is reflected both in the wide documentation of HCPs' concerns for discussing weight management with their patients and the delicate manner in which conversations around weight management unfold.^5^, ^17^, ^18^, ^19^ Although patients in the present study appeared receptive to offers of referral, accepting just over half the time a formal offer was made, this rate of acceptance is lower than previously observed in a trial where HCPs were trained to make a sensitive and effective offer of referral and three‐quarters of patients accepted.^8^ Despite the receptiveness of patients to formal offers of referral, patients did sometimes express discouragement or reference poor mental health when asked about their experience managing their weight. Notably, HCPs rarely inquired about mental health within the annual review appointment, so it was patients who created opportunities to discuss mental wellbeing in relation to their physical health. This pattern aligns with HCP beliefs that weight management can be an emotionally charged topic^17^ and indicates that patients view life factors as relevant to their achievement of healthy behaviours – a matter they would like to discuss with their HCPs.

Overall, the current study aligns with existing evidence that there is a low rate of intervention on patients living with obesity and highlights that foundational information such as measuring a patients' weight, which might reasonably take place during each annual review, is frequently omitted from these consultations. Although the wide confidence intervals reported here mean it is not possible to conclude that the NES had no impact, we found no strong evidence that the NES and its incentive affected the rate of referral for patients with obesity and hypertension and/or diabetes. While the NES aimed to mitigate barriers to weight management in England by incentivising referral to WMPs and encouraging HCPs to address weight management within patient consultations, the only difference observed here between NES and non‐NES sites was a greater emphasis on body measurements and more detailed discussions about diet in England. This difference may indicate that the NES was able to subtly impact HCPs' behaviour; however, the overall lack of impact—particularly on the frequency of discussions about weight and the rate of referral to WMPs—indicates that additional work investigating the barriers to behavioural intervention is warranted. Future work may benefit from investigating whether financial incentives (especially comparing potential gain to preventing potential loss) can overcome the barriers to intervention on patient weight over a longer timescale or whether additional training for HCPs on raising the topic of weight can effectively encourage behavioural intervention and increase referrals to weight management services.

## AUTHOR CONTRIBUTIONS

PA, SAJ, RB, AH, CA, and LH conceived and designed the study. SM, SJPH, KM, RB, SuM, AlH, and PD collected and analysed the data, overseen by PD and PA. KM created the visuals. SJPH led the writing of the manuscript. SAJ and PA supervised the writing of the manuscript. SM, RB, AH, AlH, PD, CA, SuM, LH, KM, SAJ, and PA provided feedback and edits on the manuscript.

## CONFLICT OF INTEREST STATEMENT

PA and SAJ are investigators on two publicly funded trials where Nestle donated total diet replacement products to support NHS treatment costs. In 2022, CA was a contracted qualitative methodologist for the Behavioural Insights Team (BIT) for which she was paid personally. She has worked as a consultant qualitative methodologist for Wildfowl Wetlands Trust, Linney Create, and Adelphi Real World and received personal payment.

## Supporting information


**Figure S1.** A priori scheme for coding elements of annual review consultations.
