# Supplementary material for: Effect of the National Enhanced Service for weight management on the content of annual review consultations for patients living with obesity and hypertension and/or diabetes
Source: Clin Obes. 2024 Nov 10;15(2):e12718. doi: 10.1111/cob.12718 (PMC11907094; doi:10.1111/cob.12718)
Supplement: Supplementary file 1 — Figure S1. A priori scheme for coding elements of annual review consultations. [file COB-15-e12718-s001.docx]

**Effect of the National Enhanced Service for Weight Management on the content of annual review consultations for patients living with obesity and hypertension and/or diabetes**

Stella JP Haffner MSc,^1,2^ Sarah Mounsey PhD,^3^ Rachna Begh PhD,^1^ Anisa Hajizadeh MPH,^1^ Alice E Hobson MSc,^1,2^ Paul Doody PhD,^1,2,4^ Charlotte Albury DPhil,^1^ Suzanne Mara BSc,^1^ Laura Heath MRCGP,^1^ Kayley McPherson MSc,^1^ Susan A Jebb PhD,^1,2,5,6^ & Paul Aveyard PhD ^1,2,5,6^

^1^Nuffield Department of Primary Care Health Sciences, University of Oxford, Oxford, UK

^2^NIHR Oxford and Thames Valley Applied Research Collaboration, University of Oxford, Oxford, UK

^3^School of Geography and the Environment, University of Oxford, Oxford, UK

^4^Discipline of Public Health and Primary Care, Institute of Population Health, School of Medicine, Trinity College Dublin, the University of Dublin, Ireland

^5^NIHR Oxford Biomedical Research Centre, John Radcliffe Hospital, Oxford, UK

^6^ NIHR Oxford Health Biomedical Research Centre, Warneford Hospital, Oxford, UK

**Correspondence:**

Stella JP Haffner

Radcliffe Primary Care Building, 32 Woodstock Rd, Oxford OX2 6HT

stella.haffner@phc.ox.ac.uk

*Figure S1: A priori scheme for coding elements of annual review consultations.*

| **Taking baseline measures** | | | | | | | | | | | | | |
| --- | --- | --- | --- | --- | --- | --- | --- | --- | --- | --- | --- | --- | --- |
| Are behavioural risk factors mentioned or asked about? | Does the HCP mention measured weight loss? | | Does the HCP weigh the patient? If yes, does HCP ask permission? | Does the HCP say the patient's weight out loud? | | | What interpretation does the HCP make on the patient's weight status? | | Does the HCP take any other measurements (e.g., height, waist circumference)? | Does the HCP use the word 'overweight'? | | | Does the HCP use the word 'obese'? |
| **Assessing patient’s reaction and the emotional content of the consultation** | | | | | | | | | | | | | |
| What is the patient's reaction to weighing / conversation around weight? | | Was there any sign of distress by the patient? | | | What was the emotional temperature surrounding the dialogue? | | | | | | | Is there mention of mood / emotional wellbeing? | |
| **HCP offers lifestyle management advice or resources** | | | | | | | | | | | | | |
| Does the HCP mention diet? | | | | | Does the HCP mention WMPs? | | | | | | | | |
| **How HCP talks about WMPs** | | | | | | | | | | | | | |
| Does the HCP endorse WMPs? | | | Does the HCP provide positive reinforcement to the WMP? | | | Does the HCP explain why WMPs help? | | | | | Does the HCP make a clear offer of a WMP? | | |
| **Patient reaction to WMPs** | | | | | | | | | | | | | |
| Does the HCP give the patient space to say yes or no? | | If the patient says 'yes' to a WMP, is the onus of contact given to the patient or taken up by the HCP? | | | | | | If the patient says 'yes,’ does the WMP get in touch with the patient? | | | | | |
